# Quality of life in people with chronic kidney disease: focusing on modifiable risk factors

**DOI:** 10.1097/MNH.0000000000001013

**Published:** 2024-07-22

**Authors:** Simon D.S. Fraser, Thomas Phillips

**Affiliations:** School of Primary Care, Population Sciences and Medical Education, Faculty of Medicine, University of Southampton, Southampton, UK

**Keywords:** chronic kidney disease, health-related quality of life, modifiable, risk factors

## Abstract

**Purpose of review:**

With ageing populations and rising prevalence of key risk factors, the prevalence of many long-term conditions including chronic kidney disease (CKD) is increasing globally. Health-related quality of life (HRQoL) is important to people living with CKD but not all HRQoL determinants are modifiable. This review summarizes recently identified potentially modifiable factors affecting HRQoL for people with CKD and recent trials incorporating HRQoL as an outcome.

**Recent findings:**

Considering a broad definition of ‘potentially modifiable’, many factors have been associated with HRQoL in recent observational studies. These include mental health conditions, symptoms, medications, health behaviours, weight-related issues, poor social support, lower education, limited literacy and directly CKD- related factors such as anaemia. Some potentially modifiable factors have been tested in CKD trials, though often with HRQoL as a secondary outcome, so may be underpowered for HRQoL. Interventions with evidence of effect on HRQoL include physical activity, education, some nutritional interventions and medications targeting CKD-related anaemia.

**Summary:**

Clinicians should consider the range of potentially modifiable factors influencing HRQoL as part of a holistic approach to CKD care. High-quality, adequately-powered trials, with HRQoL as a primary outcome, with interventions focusing on the other potentially modifiable factors identified are needed.

## INTRODUCTION

Chronic kidney disease (CKD) is a common but heterogenous condition with many underlying causes and wide variation in severity and risk of progression and other adverse outcomes [1,2]. The health-related quality of life (HRQoL) experienced by people with CKD also varies widely depending on many factors [3^▪▪^]. HRQoL is affected not only by CKD severity, but therapeutic interventions, particularly dialysis, and many symptoms that change as CKD progresses and play an important role in determining individual experience [3^▪▪^,^4^]. CKD also spans the age range, though is much more common in older people, and HRQoL is known to vary with age [4,5]. Multimorbidity is common among people with CKD which also significantly influences HRQoL [6]. Many aspects affecting HRQoL are not amenable to change and it is logical to focus endeavours on those that are potentially modifiable to improve HRQoL for people living with CKD. To do this, it is worth considering what we mean by ‘modifiable’ – a commonly used but poorly defined term. A potentially helpful conceptualization recommends posing a series of questions about a risk factor [7^▪^]:

(1) Is it measurable?
(2) Is it potentially changeable?
(3) Are its causes modifiable in themselves?
(4) Is it plausible as a cause?
(5) Is there empirical evidence for its effect?

The ability to infer causality from observational studies may also benefit from a framework approach to explore issues such as the causal assumptions being made [8]. In health contexts, ‘modifiable’ is often applied to individual behaviours like smoking, or clinical attributes such as anaemia, but for HRQoL a wider consideration of modifiability is important, such as the influence of health services, social support and policy. 

**Box 1:**
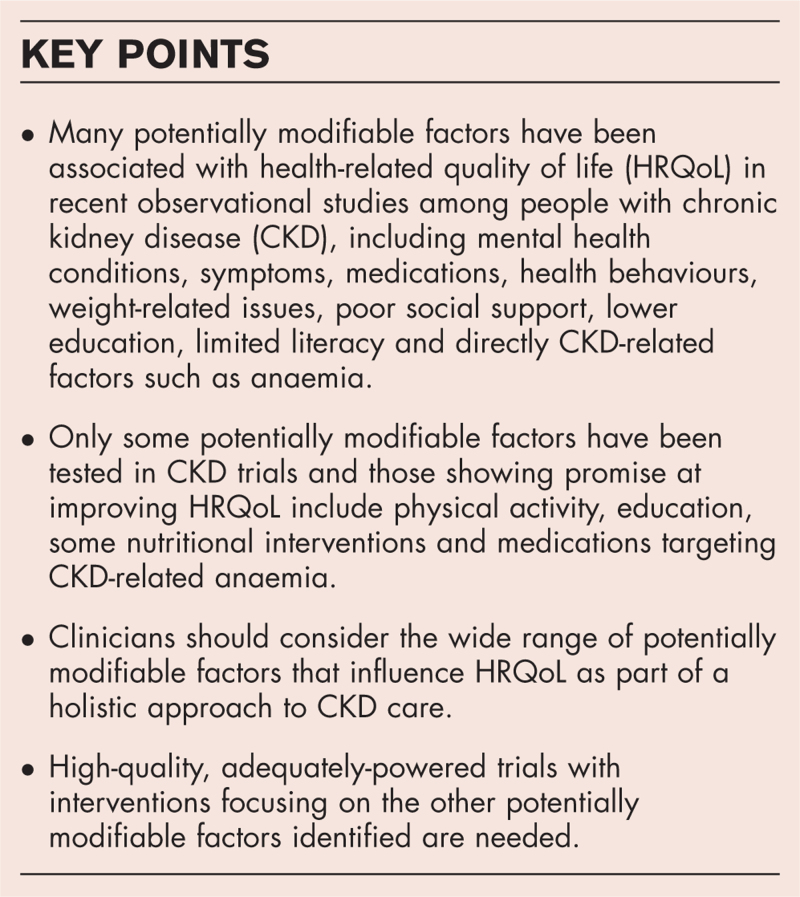
no caption available

## HEALTH-RELATED QUALITY OF LIFE

HRQoL has multiple definitions and the term is sometimes used interchangeably with ‘quality of life’ or ‘health-status’ [9]. Distilling from several definitions [10–12], HRQoL is a combination of a person's perceived functional, physical and mental health statuses. Several different outcome measures are used to quantify HRQoL in observational studies and as patient reported outcome measures in clinical trials [13]. It is beyond the remit of this review to explore every HRQoL measure, but understanding the commonly used measures and their limitations is helpful. Several are can be used in general populations but some are condition-specific. Table 1 summarizes some common measures.

**Table 1:** Summary of commonly used health-related quality of life measures

Health-related quality of life measure	Domains considered	How rated	Additional notes
EuroQoL EQ-5D [14–20]	• Mobility • Self-care • Usual activities (e.g., work, study, housework, family or leisure activities) • Pain/discomfort • Anxiety/depression Visual Analogue Scale (VAS)	Self-rated on a scale 1–3 for 3-level (3L) or 1–5 for 5-level (5L) version e.g. ‘I have no problems in walking about”, “2 – I have some problems in walking about” and “3 - I am confined to bed” Participant marks on a 0–100 scale how good or bad their health is on that day, where 100 is the best imaginable health state and 0 is the worst imaginable health state	EQ-5D responses can be used to generate an index value of health state which is usually a number between 0.0 to 1.0, where 1.0 denotes no issues in any domain and therefore ‘perfect’ health and 0.0 denotes a health state equivalent to death
12-item and 36-item short form surveys (SF-12 and SF-36) [21–23]	SF-12: Mental component summary (MCS) and physical component summary (PCS)	Comprised of twelve questions which are then collated to provide overall MCS and PCS scores on 0–100 scales	
	SF-36: eight domains; physical functioning, physical role limitations, bodily pain, general health perceptions, energy/vitality, social functioning, emotional role limitations and mental health,	Scores adjusted to be on a scale from 0–100 for each domain. MCS and PCS scores can be derived from the results in each domain [24]	
Kidney disease quality of life questionnaire (KDQOL-SF1.3) [25–27]	Questionnaire includes the SF-36 in addition to kidney specific dimensions such as symptoms of kidney disease, effects of kidney disease on daily life, burden of kidney disease, cognitive function, work status, sexual function, quality of social interaction, sleep, social support, dialysis staff encouragement and patient satisfaction	80-item measure. The SF-36 domains can be converted to provide PCS and MCS values as above.	KDQOL-SF1.3 can be converted to a kidney disease component score (KDCS) by transforming the scores to a scale of 0–100 and taking the average of all eleven kidney-specific domains. A Kidney Summary Score (KSS) was also developed to collate the kidney-specific domains of this measure
World Health Organization (WHO) measures [28,29]	WHOQOL-100	100-item measure that provides transformed scores in the domains physical health, psychological health, level of independence, social relationships, environment, spirituality, and overall HRQoL	
	WHOQOL brief version (WHOQOL-BREF)	26-item version of WHOQOL-100 and measures physical health, psychological health, social relationships and environment only, with two questions on overall HRQoL	

## RISK FACTORS ASSOCIATED WITH HEALTH-RELATED QUALITY OF LIFE IN OBSERVATIONAL STUDIES OF PEOPLE WITH CHRONIC KIDNEY DISEASE

### Mental health

A substantial body of work now demonstrates an association between mental health conditions and HRQoL. For example, baseline findings from the NURTuRE-CKD secondary care (referred) cohort of 2958 people with CKD study in the UK showed independent associations between poorer HRQoL and depression and anxiety [30^▪^]. Analysis of data from 160 patients (inpatients or at dialysis centres) using KDQoL in a German cohort identified depression as an independent predictor of worse physical component summary (PCS) and mental component summary (MCS) [31]. Depression was also among several independent associations with lower HRQoL among 649 nondialysis CKD patients in the nationally-representative US medical expenditure panel survey (MEPS) [32].

Changes over time in HRQoL were evaluated in the ‘Screening for Chronic Kidney Disease among Older People across Europe’ (SCOPE) project, a multicentre 2-year prospective cohort involving people over 75 years attending outpatients clinics in Austria, Germany, Israel, Italy, the Netherlands, Poland, and Spain, with 1748 participants completing the EQ-5D-5L. Those with greater Geriatric Depression Scale-Short Form scores were more likely to show EQ-VAS decline over time [33].

Similarly, in a German population-representative study of 5159 adults using the SF-36, depression was negatively associated with both PCS and MCS scores. However, while poorer kidney function was associated with most dimensions relating to physical HRQoL, there was no clear association for mental HRQoL [34]. An Irish study among 268 people with a range of CKD severities (including nondialysis dependent CKD, those using different dialysis modalities and transplant recipients) using SF12 and the Hospital Anxiety and Depression Scale (HADS) showed that mental health conditions were common with depression, anxiety and having a mental health diagnosis all associated with lower HRQoL [35]. A small cross-sectional study among people with nondialysis dependent CKD tested for cognitive function, frailty and HRQoL also showed that cognitive impairment was associated with poorer HRQoL (SF-36) [36].

### Education and work

Several recent studies have demonstrated association between education, work and HRQoL in a variety of settings. Baseline analyses from the NURTuRE-CKD cohort found that those with lower educational attainment were more likely to report poorer HRQoL and that being in work was associated with better HRQoL [30^▪^]. A cross-sectional study in secondary care setting in Nigeria including 220 people with CKD stages 1 to 4 used the 15-dimensional HRQoL questionnaire and showed that lower education level and unemployment or self-employment were independently associated with poor HRQoL [37]. A cross-sectional study of 300 people with CKD at two Ethiopian hospitals using KDQoL-36 identified that poorer PCS was independently associated with lower levels of literacy [38].

### Illness denial and patient activation

A survey of 14 renal units in England of 3013 people with nondialysis dependent CKD, dialysis, and kidney transplant, used latent class analysis to determine HRQoL and symptom burden subgroups. Lower patient activation levels were associated with higher symptom burden and reduced HRQoL across CKD stages and treatment modalities [39]. An interesting cross-sectional study of 100 people with CKD in an outpatient setting in a single hospital in Italy investigated the link between illness denial, specific personality traits (‘Big-Five’) and HRQoL using the KDQOL-SF. Illness denial was associated with better HRQoL and certain personality traits (extraversion, agreeableness, conscientiousness, neuroticism and openness) were associated with better HRQoL in certain domains [40]. A Dutch cohort study of 180 older adults with eGFR ≤20 ml/min/1.73 m^2^ assessed apathy symptoms using a subscale of the Geriatric Depression Scale. Apathy was common (36% of older patients with CKD) and presence of apathy symptoms was associated with lower physical and cognitive functioning and HRQoL at baseline [41].

### Health behaviours

Physical activity has been linked to HRQoL in several observational studies. An analysis of step count among 558 adults with CKD suggested that walking between 7000 and 12 000 steps daily was associated with high HRQOL and step count demonstrated an inverse U-shaped relationship with SF-36 subscale scores; lower than 7000 was associated with lower PCS and MCS scores and higher than 12 000 with lower MCS score [42]. Lower physical activity was also associated with worse HRQoL in the MEPS study described above [32].

A propensity score matching approach was used among 1618 patients from the KNOW-CKD study to estimate the effect of physical activity on HRQoL. ‘Health-enhancing physical activity’ (150 min of moderate-intensity or 75 min of vigorous-intensity aerobic physical activity a week) was associated with better HRQoL [43].

Smoking has been implicated in worse HRQoL. The NURTuRE-CKD HRQoL study found that being an ex-smoker was associated with worse HRQoL by EQ-5D-3L mapped index value [30^▪^]. The cross-sectional Ethiopian study mentioned above also found previous smoking to be independently associated with poorer PCS [38].

Diet, specifically low protein diet, was associated with both depression and poor HRQoL (EQ-5D-5L index) after adjusting for relevant confounders in a cross-sectional study of 571 people with CKD in South Korea [44]. In a secondary analysis from a randomized trial of an exercise intervention in 99 people with CKD stage 3b-4, poor appetite was a component of baseline ‘geriatric syndromes’ (including cognitive impairment, poor appetite, dizziness, fatigue, and chronic pain) that were associated with lower HRQOL (SF-36 and EQ-5D-5L) [45]. Among 100 people with autosomal dominant polycystic kidney disease in a cross-sectional study, a positive relationship was observed between dietary adherence and HRQoL (EQ-5D-3L) [46].

### Medications

The NUTuRE-CKD HRQoL study identified that polypharmacy was independently associated with worse overall HRQoL and problems in most dimensions, possibly linked to greater comorbidity. Some individual medications were also associated with HRQoL measures. Taking prednisolone was associated with worse HRQoL in the self-care dimension. In contrast, treatment with renin-angiotensin system inhibitors was associated with fewer reported problems in mobility and usual activities dimensions [30^▪^].

Polypharmacy was also negatively associated with HRQoL in the MEPS study [32]. Over two-thirds had ‘major polypharmacy’ (5–9 medication classes), and ‘hyperpolypharmacy’ (≥10 medication classes). Mean PCS score was lower among those with major compared to minor polypharmacy. Age, income, health insurance coverage, lower physical activity, census region, number of comorbidities, depression, diabetes, arthritis, and cardiovascular disease were also negatively associated with HRQoL [32].

### Weight

Several studies have identified associations between overweight and obesity and lower HRQoL. For example, in the German population-based study described above, higher BMI, was associated with lower perceived general state of health [34]. This was also the case in the NURTuRE-CKD HRQoL baseline analyses, where obesity was independently associated with poorer HRQoL [30^▪^].

### Symptoms

A major systematic review and meta-analysis including 449 studies and a total of 199 147 participants from 62 countries identified the high symptom and HRQoL burden experienced by people with CKD [3^▪▪^]. Fatigue, poor mobility, drowsiness, and pain (especially bone or joint pain) were particularly common. HRQoL was reported in 361 of the studies confirming that HRQoL scores were lowest in people on dialysis, better for those receiving a kidney transplant and higher for those not requiring kidney replacement therapy [3^▪▪^].

Other recent studies exploring symptoms and HRQoL have identified the following:

(1) Pain and frailty in NURTuRE-CKD HRQoL [30^▪^].
(2) ‘Geriatric syndromes’ (cognitive impairment, poor appetite, dizziness, fatigue, and chronic pain) in secondary analysis of a randomized trial of an exercise intervention [45].
(3) Reduced physical function and physical performance in a cross-sectional study of 61 older people with CKD stages 3–5 in Japan and in the SCOPE study described above [33,47].
(4) Sleep disorders in a cross-sectional study of 172 people with nondialysis CKD [48].
(5) Constipation in a systematic review and meta-analysis exploring gastrointestinal symptoms among people with nondialysis CKD [49].
(6) Difficulty with usual activities, drowsiness and shortness of breath in a UK cross-sectional study among 216 people with conservatively-managed CKD stage 5. Variables independently associated with poorer EQ-VAS were difficulty performing usual activities, self-rated drowsiness and shortness of breath [50].

### Kidney function

Several studies have linked kidney function with HRQoL. For example, in the population-representative German study, poorer kidney function was associated with most dimensions relating to physical HRQoL while for mental HRQoL there was no clear association with different eGFR categories [34]. In NURTuRE-CKD HRQoL, higher eGFR was independently associated with a higher self-reported VAS score [30^▪^]. By contrast, the SCOPE study findings suggested that decrease in kidney function did not contribute to EQ-VAS decline over a two-year period in early CKD [33]. Rapid kidney function decline was, however, linked to rapid HRQoL deterioration in the KNOW-CKD study (610 participants with nonrapid decline and 360 with rapid decline). The PCS score decreased significantly in both rapid and nonrapid decline groups, while the MCS score decreased significantly only in the rapid kidney function decline group [51].

### Potentially modifiable conditions closely linked to chronic kidney disease

A cross-sectional study of 423 10-year long term survivors in the Frontier of Renal Outcome Modifications in Japan study found that baseline systolic blood pressure and history of hyperuricemia were predictors of HRQOL [52]. Similarly in the population-representative German study, hypertension was associated with lower perceived general state of health, as was heart failure [34].

Several studies identify anaemia as contributing to poor HRQoL. These include a cross-sectional Sri Lankan study in 886 people with CKD of varying severity using a structural equation modelling approach to identify factors contributing directly or indirectly to HRQoL (EQ-5D-3L) [53]. Symptoms were strongly negatively associated with HRQoL and decreased kidney function, lower haemoglobin and greater number of comorbidities directly contributed to increased symptoms, therefore indirectly influencing HRQoL. The NURTuRE-CKD HRQoL study identified haemoglobin <100 g/l as independently associated with worse HRQoL [30^▪^]. An online US survey of 410 patients and 258 care partners exploring the burden experienced by people with anaemia and CKD found that patients with anaemia reported lower average HRQoL and partners reported severe burden [54].

### Studies among people requiring kidney replacement therapy

From studies among people requiring kidney replacement therapy (KRT), there are many similar themes to the nondialysis dependent population. These are summarized in Table 2.

**Table 2:** Exposures associated with worse health-related quality of life among people requiring kidney replacement therapy

Exposure associated with worse HRQoL	Study /studies
Pruritis	• A systematic review and narrative synthesis of 18 studies exploring the relationship between CKD-associated pruritis and HRQoL in people receiving haemodialysis. CKD-associated pruritis severity was associated with worsening of HRQoL, potentially partially mediated by sleep disturbance [55] • A retrospective cross-sectional study of 6221 patients in 152 renal clinics in multiple European countries. HRQoL was assessed using KDQoL and pruritis using the 5-D Itch questionnaire. Prevalence of pruritis was high (about 48%) and both mental and physical HRQoL score were lower with greater pruritis severity [56].
Limited health literacy	• A longitudinal study of 413 people at dialysis units in Slovakia describing three health literacy groups (low, moderate and high) using the Health Literacy Questionnaire and assessing HRQoL using KDQoL short form found that patients with low health literacy had poorer HRQoL at baseline compared to high-HL patients but no significant associations of lower HL with the deterioration of mental or physical HRQoL over 2 years [57]
Not being able to work	• A study of 517 haemodialysis patients with hypertension using EQ-5D-5L at baseline and two follow up points. Not being able to work was associated with lower HRQoL as was BMI and salt intake [58].
Physical inactivity	• The same study of 517 haemodialysis patients with hypertension showed that not exercising was associated with lower HRQoL as was BMI and salt intake [58] • A study of 130 patients on haemodialysis and peritoneal dialysis identified generally low physical activity but higher physical activity levels were associated with better HRQoL in both groups [59]
Low body mass index	• The study of 517 haemodialysis patients with hypertension showed lower HRQoL associated with lower body mass index [58] • A cross-sectional study in Ethiopia among haemodialysis patients at eleven dialysis centres also showed lower body mass index (<18.5) associated with low HRQOL [60]
Frailty	• A cross-sectional study in 93 haemodialysis patients in New Zealand showed that frailty was associated with worse HRQoL [61]
Poor social support	• The cross-sectional study in Addis Ababa at eleven dialysis centres showed poor social support to be associated with worse HRQoL [60]
Poor medication adherence	• The same Ethiopian study found poor medication adherence to be associated with worse HRQoL [60]
Low educational attainment	• The same Ethiopian study found poor no formal education to be associated with worse HRQoL [60]
Mode of dialysis	• A prospective cohort of 109 patients using different dialysis modalities using KDQOL-SF showed that crude physical composite summary of HRQoL was higher in those choosing home dialysis but mental composite summary was similar across groups. After adjustment, patients choosing home dialysis had improved mental composite summary over time compared to those selecting in-centre haemodialysis or conservative care [62] • A Cross-sectional study in South Africa using KDQOL-SF36 to assess HRQoL among 150 patients (50 each using haemodialysis, peritoneal dialysis and conservative care). Physical composite, role–physical, vitality, and emotional well being scores were poorer in dialysed patients. Physical composite summary, pain, vitality, and social functioning KDQOL scores were poorer in PD compared to HD [63]
Frequency and duration of dialysis (worse HRQoL with greater frequency)	• The Ethiopian study described above found greater frequency of dialysis (more than two sessions per week) and longer duration of haemodialysis treatment (≥ 12 months) associated with low HRQOL [60]

CKD, chronic kidney disease; HRQoL, health-related quality of life.

### Studies among children with chronic kidney disease

A systematic review and meta-analysis of 14 studies among 5- to 18-year-old patients with kidney failure (using PedsQL 4.0 Generic Core Scale (GCS) and the PedsQL 3.0 ESRD Module) identified that kidney transplant patients reported a significantly higher HRQoL than those on dialysis [64]. Those on peritoneal dialysis reported better HRQoL than those on haemodialysis. A longitudinal study involving 692 children (median age 11.2, median 8.3 years CKD duration) using PedsQL found that longer CKD duration was associated with better HRQoL on child self-report [65]. The authors’ had expected disease progression or worsening CKD to be associated with worsening HRQoL and suggested their findings may represent a degree of adaptation by children with CKD [65]. A cohort study in Australia and New Zealand assessed trajectories of HRQoL among 377 children with CKD aged 6–18 years over four years. The authors concluded that improvement in HRQoL over time for children on dialysis was likely related to transition to transplantation. Children with CKD stage 1–5 and transplant recipients at baseline experienced stable HRQoL over time [66].

## RECENT TRIALS WITH HEALTH-RELATED QUALITY OF LIFE AS AN OUTCOME MEASURE

Relatively few of these wide-ranging potentially modifiable factors have been tested in CKD trials. Table 3 summarizes recently conducted trials that have included HRQoL as either primary or secondary outcome [67^▪▪^,^68^,^69^,^70^,71^▪^,^72^–^74^,^75^,76^▪^,^77^,^78^].

**Table 3:** Recent trials in people with CKD that included HRQoL as an outcome measure

Intervention type	First author of study and year of publication	Country	Number of total participants	CKD stages included	Baseline mean ± SD/median (IQR) eGFR of total participants (ml/min/1.73 m^2^)	Intervention	Comparator	Blinding	Follow-up duration	Primary outcome	HRQoL as primary or secondary outcome	HRQoL measure(s) used	Evidence of HRQoL benefit
Physical activity	Greenwood 2024 [67^▪▪^]	UK	340	G2–5	53.8 (13.5)	Kidney BEAM physical activity digital health intervention	Waiting list control	Single blinded	3 months	HRQoL	Primary	KDQoL-SF1.3	Significant improvement in MCS for intervention group
Physical activity	Bohlke 2022 [68]	Brazil	150	G3a-4	63.4	Aerobic and resistance training	Usual care	Unblinded	32 months	Survival	Secondary	SF-36	No significant effect
Physical activity	Thompson 2022 [69]	Canada	44	G3b-4	28 (21, 37)	In-centre and home-based exercise programme	Usual care	Unblinded	6 months	Blood pressure	Secondary	SF-12, EQ-5D	No significant effect
Education	Sarker 2022 [70]	Bangladesh	126	G1–3b	N/A	Education and home visits for BP monitoring	Usual care	Unblinded	6 months	scores on the Chronic Kidney Disease Knowledge Questionnaire	Secondary	EQ-5D-5L	No significant effect
Education	Lee 2022 [71^▪^]	Taiwan	76	G3b-5	36.9 ± 20.8	Patient self-management education sessions	Usual care	Single blind	6 months	HRQoL	Primary	SF-12	Significant improvement in MCS and PCS for intervention group
Medications aimed at slowing CKD progression	Weir 2023 [72]	Canada	533	G3a-4	33 (12)	Micro-particle curcumin	Placebo	Double-blind	6 months	eGFR and albuminuria	Secondary	SF-36	No significant effect
Medications aimed at slowing CKD progression	Cha 2022 [73]	Korea	150	G3a-4	33.8 ± 12.5	AST-120	Usual care	Unblinded	11 months	Gait speed	Secondary	KDQOL-36	Significant improvement in ’quality of social interaction’ domain only in intervention group
Medication for depression	Saleh-Arong 2022 [74]	Thailand	53	G3a-5	18.3 ± 16.3	Argomelatine	Usual care	Unblinded	2 months	Depression score	Secondary	WHO-QOL-BREF	No significant effect
Medications to treat CKD-related anaemia	Greenwood2023 [75]	UK	75	G3a-4	35 ± 12	Ferric carboxymaltose	Placebo	Double-blind	3 months	6-min walk test	Secondary	KDQOL-36	No significant effect
Medications to treat CKD-related anaemia	Johansen 2023 [76^▪^]	Global	614	G3a-5	N/A	Daprodustat	Placebo	Double-blind	7 months	Change in haemoglobin	Secondary	SF-36	Significant improvement in ’vitality’ domain for intervention group
Nutrition	Hamidianshirazi 2023 [77]	Iran	120	G3a-4	32.3 ± 1.6	Diet therapy and nutritional education	Usual care	Unblinded	6 months	HRQoL	Secondary	SF-12	No significant effect
Nutrition	Hosojima 2022 [78]	Japan	102	G3a-4 & A2	N/A	Low protein food replacement	Usual care	Unblinded	6 months	Estimated dietary protein intake	Secondary	KDQOL-36	Significant improvement in 'social support’ domain for intervention group

CKD, chronic kidney disease; HRQoL, health-related quality of life.

Benefit to HRQoL in at least one study was shown for education interventions, physical activity interventions, medications to treat CKD-related anaemia, and nutritional interventions. No benefit to HRQoL was shown for medications aimed at slowing CKD progression and medications for depression. Of the twelve trials in Table 3, it is notable that only two included HRQoL as a primary outcome measure [67^▪▪^,71^▪^].

**FIGURE 1:**
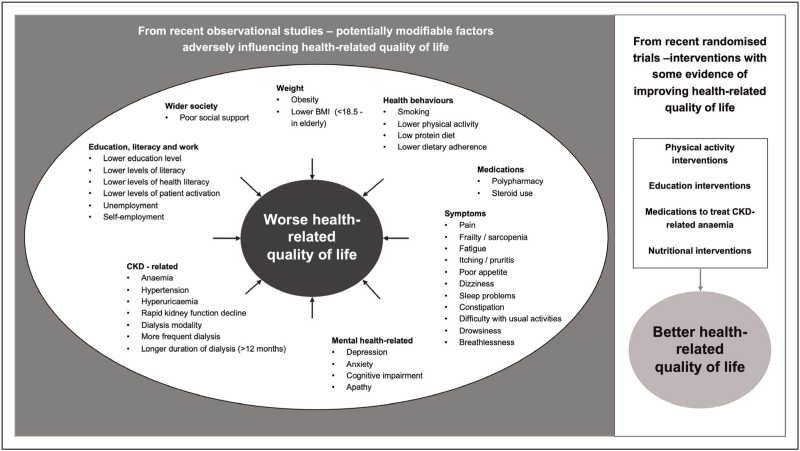
Potentially modifiable factors known to independently affect health related quality of life among people with CKD and interventions with evidence of effectiveness. CKD, chronic kidney disease.

Figure 1 provides an overview, summarizing the potentially modifiable factors associated with HRQoL in recent observational studies and those tested as interventions with some evidence of effect in recent trials.

Follow-up analysis of an older trial (excluded from Table 3) of gastric bypass among people with diabetic kidney disease and obesity showed evidence of HRQoL improvement as a secondary outcome [79].

## CONCLUSION

Poor HRQoL is common among people with CKD and many potentially modifiable determinants have been identified, including mental health conditions, symptoms, medications, health behaviours, weight-related issues, poor social support, lower education, limited literacy and directly CKD-related factors such as anaemia. Only some of these have been intervention targets in CKD trials with HRQoL as an outcome. Promising interventions for improving HRQoL include physical activity, education, some nutritional interventions and medications targeting CKD-related anaemia. Clinicians should consider the wide range of potentially modifiable factors that influence HRQoL as part of a holistic approach to CKD care. High-quality, adequately-powered trials, using HRQoL as a primary outcome, with interventions focusing on the other potentially modifiable factors identified are needed.

## Acknowledgements

*None.*

### Financial support and sponsorship

*The authors received no funding for this work. The NURTuRE CKD HRQoL study is funded by a Kidney Research Funding UK (KRUK) research grant in 2022 (RP/005/20210728).*

### Conflicts of interest

*There are no conflicts of interest.*

## REFERENCES AND RECOMMENDED READING

Papers of particular interest, published within the annual period of review, have been highlighted as:

▪ of special interest
▪▪ of outstanding interest
